# Reverse transcription loop-mediated isothermal amplification assay for rapid detection of Bovine Rotavirus

**DOI:** 10.1186/1746-6148-8-133

**Published:** 2012-08-15

**Authors:** Zhixun Xie, Qing Fan, Jiabo Liu, Yaoshan Pang, Xianwen Deng, Zhiqin Xie, Xie Liji, Mazhar I Khan

**Affiliations:** 1 Department of Biotechnology, Guangxi Veterinary Research Institute, 51 You Ai Road, Nanning, Guangxi, 530001, China; 2 Guangxi Key Laboratory of Animal Vaccines and Diagnostics, 51 You Ai Road, Nanning, Guangxi, 530001, China; 3 Department of Pathobiology & Veterinary Science, University of Connecticut, Storrs, CT, 06260-3089, USA

**Keywords:** BRV, VP6 gene, Reverse transcription loop-mediated isothermal amplification (RT-LAMP)

## Abstract

**Background:**

Bovine rotavirus (BRV) infection is common in young calves. This viral infection causes acute diarrhea leading to death. Rapid identification of infected calves is essential to control BRV successfully. Therefore development of simple, highly specific, and sensitive detection method for BRV is needed.

**Results:**

A reverse transcription loop-mediated isothermal amplification (RT-LAMP) assay was developed and optimized for rapid detection of BRV. Specific primer sets were designed to target the sequences of the VP6 gene of the neonatal calf diarrhea virus (NCDV) strain of BRV. The RT-LAMP assay was performed in a water bath for 60 minutes at 63°C, and the amplification products were visualized either directly or under ultraviolet light. This BRV specific RT-LAMP assay could detect 3.32 copies of subtype A BRV. No cross-reactions were detected with other bovine pathogens. The ability of RT-LAMP to detect bovine rotavirus was further evaluated with 88 bovine rectal swab samples. Twenty-nine of these samples were found to be positive for BRV using RT-LAMP. The BRV-specific-RT-LAMP results were also confirmed by real-time RT-PCR assay.

**Conclusions:**

The bovine rotavirus-specific RT-LAMP assay was highly sensitive and holds promise as a prompt and simple diagnostic method for the detection of group A bovine rotavirus infection in young calves.

## Background

Rotavirus, a double-stranded RNA virus, is a member of the family Reoviridae. Rotaviruses are classified into six, or possibly seven serogroups [1,2]. Rotavirus infections with group A are the major cause of acute diarrhea among newborn animals and humans leading to death [3,4]. Identification of infected calves, in combination with a proper vaccination program, is essential to control BRV successfully. The current methods for the detection and characterization of BRV, which include virus isolation, immunoassay, electron microscopy, and nucleic acid hybridization, are time consuming and laborious [5-7]. On the other hand, the polymerase chain reaction (PCR) has been used successfully for the detection and characterization of BRV [8-10]. Unfortunately, PCR assays require sophisticated equipment, which is costly to maintain, and must be performed in specialized laboratories (Additional file 1: Figure S1 and S2).

The recently described loop-mediated isothermal amplification (LAMP) can amplify specific target DNA sequences with high sensitivity and can be completed within 60 minutes under isothermal conditions without the need of a thermal cycler and specialized laboratory [11]. This technique eliminates the heat denaturation step for DNA synthesis used in conventional PCR, and relies instead on auto-cycling strand displacement DNA synthesis achieved by a DNA polymerase with high strand displacement activity and a set of two specially designed inner and two outer primers. Another important feature of LAMP is a resulting color change following the addition of a fluorescent dye, making it visible to the naked eye. The LAMP assay has been used successfully to detect many pathogens [12-19] and in using reverse transcriptase, it has been further adapted for the detection of RNA viruses [15,16]. The objective of the study reported here was to develop and optimize the reverse transcription LAMP (RT-LAMP) assay for the detection of group A BRV in calves.

## Methods

### Cells and Virus Strains

Table 1 lists the pathogen strains used in this study and describes the following: ten group A BRV strains, five other bovine pathogens other than BRV, four negative controls, three normal bovines rectal swab samples (one each from normal bovine nasal mucus and blood sample). Nasal mucus swabs and blood samples were taken from all bovine following routine pre slaughter examination for the normal animals to be slaughter. The collection of these samples could be considered as part of regular and routine examination, therefore no official review and approval of the Guangxi Veterinary Research Institute was needed. The viruses were propagated using rhesus monkey epithelial cell line (MA-104) and grown in Eagle’s minimal essential medium (MEM) according to previously described methods [6]. MEM was supplemented with 10% fetal calf serum free from BRV and BRV antibodies (Shijiqing, China). The BRV strains were titrated by plaque assay in MA-104 cells in accordance to the published protocols [6,16]. 

**Table 1:** Pathogens used and RT-LAMP assay results

		**Source**	**RT-LAMP result**	
			**Agarose gel electrophoresis**	**Color change after adding dye**
**Bovine rotavirus (BRV)** NCDV	genotype G6P[1]	CVCC	+	+
BRV014	G6P[5]	CVCC	+	+
GX-BRV-1	G6P[11]	GVRI	+	+
GX-BRV-2	G6P[5]	GVRI	+	+
GX-BRV-3	G10P[11]	GVRI	+	+
GX-BRV-4	G6P[11]	GVRI	+	+
GX-BRV-5	G6P[11]	GVRI	+	+
GX-BRV-6	G6P[5]	GVRI	+	+
GX-BRV-7	G6P[5]	GVRI	+	+
GX-BRV-8	G10P[11]	GVRI	+	+
**Other bovine pathogens**				
Bovine virus diarrhea (BVDV)		CVCC	-	-
*Mycobacterium bovis* (MB)		GVRI	-	-
Classical swine fever virus (CSFV)		CVCC	-	-
Infective bovine rhinotracheitis virus (IBRV)		CVCC	-	-
Bovine Coronavirus (BCV)		GVRI	-	-
**Negative control**				
Rectal swab of normal bovine1		GVRI	-	-
Rectal swab of normal bovine2		GVRI	-	-
Rectal swab of normal bovine3		GVRI	-	-
Nasal swab of normal bovine		GVRI	-	-
Blood of normal bovine		GVRI	-	-
**cells**				
cell −1		GVRI	-	-
cell −2		GVRI	-	-
cell −3		GVRI	-	-
Cell-4		GVRI	-	-
cell −5		GVRI	-	-

CVCC = China Veterinary Culture Collection Center.

GVRI = Guangxi Veterinary Research Institute.

Positive = +; Negative = −.

### DNA/RNA Extraction

Genomic viral RNA, which includes BRV and other bovine virus strains from sample cultures, was extracted from infected MA-104 cell culture supernatant, as well as from swab sample cells from normal bovine using the TRIZOL RNA extraction kit in accordance with the manufacturer’s protocol (Invitrogen, Carlsbad, CA, USA). Mycobacterium DNA was extracted using phenol: chloroform isoamyl alcohol (1:1:24 v/v) according to the Gibco BRL manufacturer’s protocol (Gibco BRL, Grand Island, New York, USA). All nucleic acid samples were stored at −70°C until use.

### Design of group A specific BRV RT-LAMP primers

**Figure 1:**
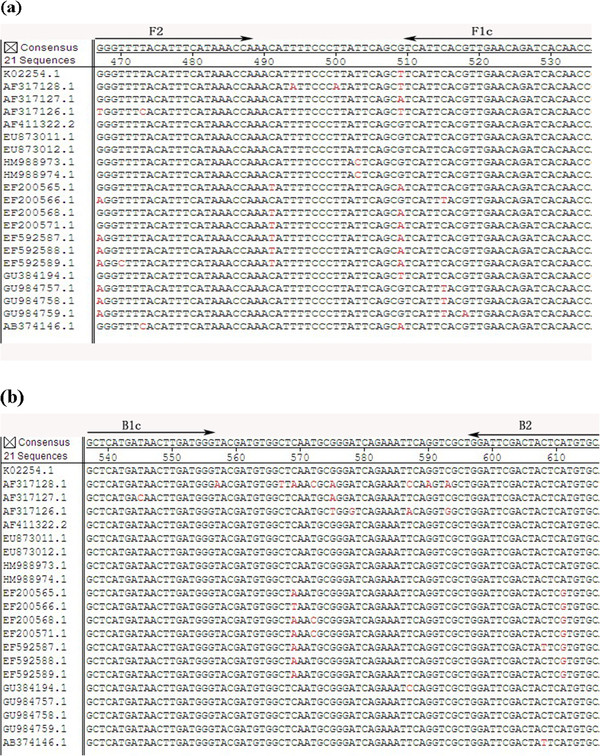
**(a and b).** Multiple sequence alignment of BRV VP6 genes.

Primer design for Group A specific bovine rotavirus- RT-LAMP was based on the published sequence of strain neonatal calf diarrhea virus (NCDV) (GenBank, accession no. K02254.1). The NCDV sequence was aligned with the available sequences of 21 isolates (Figure 1 a & b) to identify the conserved regions using Primer Explored V4 soft ware available from: http://primerexplorer.jp/e/. A set of six primers comprised of two outer, two inner, and two loop primers was designed and is shown in Table 2. The inner primers, which are known as the forward inner primer (FIP) and the backward inner primer (BIP), each have two distinct sequences corresponding to the sense and antisense sequences of the target, one for priming the first stage, and the other for self-priming in later stages in the reaction. FIP contains F1c (complementary to F1), and the F2 sequence. BIP contains the B1c sequence (complementary to B1), and the B2 sequence. The outer primers (F3 and B3) were used in the initial steps of LAMP reactions, but later during the isothermal cycling only the inner primers were used for strand displacement DNA synthesis. Two additional loop primers (loopF and loopB) were designed to accelerate the amplification reaction as described [18]. The sequences of the selected primers were compared to VP6 gene sequences [20-22]. All the primers were synthesized and purified by Invitrogen Inc (Guangzhou, China). 

**Table 2:** Details of oligonucleotide primers used for RT-LAMP assay

**Primer name**	**sequence(5’-3’)**	**Genome position**
FIP_a_ = F2 + F1c	GTTGTGATCTGTTCAACGTGAATGA-gaattc-GGGTTTTACATTTCATAAACCAA	F1c,510-534 F2,467-489
BIP_b_ = B2 + B1c	GCTCATGATAACTTGATGGG- gatatc-GCACATGAGTAGTCGAATCC	B1c,537-556 B2,597-616
F3	TTGCAAAATAGAAGACAAAGAAC	444-466
B3	GTTGCGTATTAGCTGGCG	625-642
LoopF	CTGAATAAGGGAAAATGTTTGGTTT	483-507
loopB	GATGTGGCTCAATGCGGG	560-577

Genome position according to the bovine rotavirus complete genome sequence (GenBank accession no. K02254.1).

_a_ FIP is composed of F2 and F1c, and they are linked by a sequences of EcoRIof -gaattc-.

_b_ BIP is composed of B2 and B1c, and they are linked by a sequences of EcoRV of -gatatc-.

### Real-time RT-PCR

The sensitivity of the RT-LAMP method was compared to real-time RT-PCR using the two primers and one of the following probes: F5 (5’- TCATTTCAGTTGATGAGACCACC -3’), F6 (5’- ATTCAATTCTAAGCGTGAGTCCTAC -3’), or HEX-AATATGACACCAGCGGTAGCGGC- BHQ1. This real time RT-PCR amplifies a 112-bp target sequence of the VP6 gene of group A BRV [10]. Real-time RT-PCR amplification was carried out using the Real-time one step RT-PCR Kit (Takara, Dalian, China) as described in previously published protocol (10). After the real-time RT-PCR was performed, cycle threshold (CT) was manually setup to reflect the best kinetic PCR parameters, such that any nonspecific amplification in reaction could be analyzed.

### Optimization of the RT-LAMP condition

The RT-LAMP assay was optimized using various concentrations of primers, buffers, salt and RNA/DNA templates for positive and negative controls. The reaction mixture was optimized to 25 ul, containing primers in various concentrations, 1.4 mM of each deoxyribonucleotide triphosphate, 0.8 M of betaine (Sigma Chemical Co., Beijing, China), 2.5 ul of 10 × Thermo buffer, 8 mM MgSO4, 8 U of *Bst* DNA polymerase (large fragment; New England Biolabs), 0.125U of enhanced Avian myeloblastosis virus reverse transcriptase, and 2 ul of the extracted target RNA. To determine the optimal duration for the RT-LAMP assay, the primer and reverse transcribed sample mixtures were incubated in a 63°C water bath for 20, 40, 60, and 80 minutes. At the end of each incubation period, the reaction was terminated by heating at 80°C for 5 minutes. The reaction temperature was optimized using 61°C, 62°C, 63°C, and 64°C.

### Analysis of RT-LAMP product

In order to analyze the amplified products, three detection methods were evaluated. First, turbidity: the accumulation of magnesium pyrophosphate, a byproduct of the DNA amplification reaction, increases the turbidity of the sample. Turbidity was evaluated by visual inspection of the samples, comparing them to a negative control sample. Second, color change: 1 ml of 10,000 × SYBR Green I nucleic acid stain was added to the tube after the reaction. Samples turning yellow-green were considered positive, while samples turning orange were negative. Samples showing fluorescence under an ultraviolet hand lamp at a 365-nm wavelength were considered positive as described previously [12]. Samples were compared with a negative control to account for background fluorescence. Third, gel electrophoresis: run on a 1% agarose gel, RT-LAMP reaction end product yields a combination of DNA fragments of varying sizes [11]. Therefore, the presence of a smear or a pattern of multiple bands of different molecular weights indicates a positive result. A molecular marker was used to estimate amplified product size.

Lastly, a restriction enzyme analysis: EcoRI and EcoRV restriction of the RT-LAMP product confirms reaction specificity [14]. Briefly, digestion reactions were performed using 3 ul of RT-LAMP product with 12 U of restriction enzyme in 25 ul total volume. The size of the digested fragments was estimated by agarose gel electrophoresis as described [19].

### Evaluation of RT-LAMP

To evaluate specificity, the RT-LAMP test was performed on a panel of viral isolates from bovine’s reference viruses, (Table 1). The panel included three rectal and one nasal swab samples from a normal bovine, one blood sample from a normal bovine, one cell sample (repeated five times) as a negative control, ten strains of BRV, and five different bovine DNA and RNA pathogens (Table 1).

**Figure 2:**
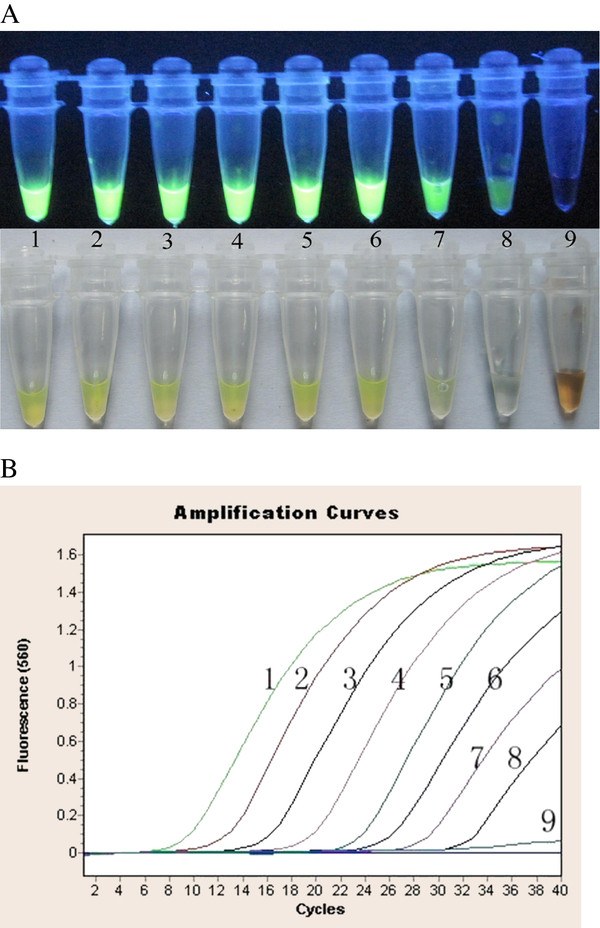
**Sensitivity of RT-LAMP-and real-time RT-PCR.** (**A**) RT-LAMP product; (**B**): real-time RT-PCR,1: 3.32 × 10^7^copies/tube, 2: 3.32 × 10^6^copies/tube, 3:3.32 × 10^5^copies/tube,4: 3.32 × 10^4^copies/tube, 5: 3.32 × 10^3^copies/tube; 3.32 × 6:10^2^copies/tube, 7: 3.32 × 10^1^copies/tube,8: 3.32 × 10^0^copies/tube; 9: 3.32 × 10^-1^copies/tube.

The detection limit of RT-LAMP was tested and compared with real-time RT-PCR using triplicate templates at identical concentration. RNA transcripts corresponding to the VP6 of NCDV strain were generated for use as standards in the analysis of sensitivity of the assay. Briefly, RNA was extracted from NCDV strain using the TRIZOL RNA extract reagent. The purified RNA was resuspended in distilled water and used in the RT-PCR reaction. The amplified product of VP6 was cloned into the pGM-T vector (TaKaRa, Dalian, China) according to the manufacturer’s directions and sequenced to verify its identity. The recombinant plasmid pGM-T-VP6 was linearized by digestion with restriction enzyme NotI, gel purified, and used as a template with a Ribo Max T7 In Vitro Transcription System (Promega, Madison, Wisconsin, USA) according to the manufacturer’s protocol. The length of RNA transcripts was verified by agarose gel electrophoresis. The RNA of VP6 was quantitated using UV spectrophotometry at 260 nm, and calculated copy numbers were calculated from the concentration as described previously [20]. A series of 10-fold dilutions were used to test the assay’s sensitivity of BRV RT-LAMP (Figure 2). 

### Detection of clinical specimen by RT-LAMP assay

After validation studies, we determined the reliability of the group A specific BRV-RT-LAMP as a method of viral RNA detection for clinical specimens. Written informed consent was obtained from each participating farm owner. On participating farms, the veterinarian collected rectal swab samples from the calves. The rectal swab samples were taken from calves between 3 to 180 days age, and were considered as part of regular and routine clinical-diagnostic care. No official review and approval of Guangxi Veterinary Research Institute was needed. A total of 88 rectal swab samples were collected from calves with acute diarrhea from different dairy farms in the Guangxi province (Table 3). The samples were placed into 1 ml of sterilized water, and processed as described previously [12]. BRV-specific RT-LAMP assay was performed as described above. The results of group A specific BRV RT-LAMP were compared with the results of BRV real-time RT-PCR. Restriction enzyme analysis of RT-LAMP products and its sequencing were used to assess the reliability of the methods for the rapid detection of BRV. 

**Table 3:** Comparison of real-time RT-PCR and RT-LAMP for the detection of BRV in clinical samples

**Location of samples**	**Number of Samples**	**Number of positive samples for assay**		
		**Real-time RT-PCR**	**RT-LAMP**	**Sequencing**
Nanning	24	7	7	correct
Liuzhou	15	3	3	correct
Fangcheng	10	3	3	correct
Shangsi	13	5	5	correct
Guilin	20	10	10	correct
Hengxian	16	1	1	correct
Total	88	29	29	correct

^a^ RT-LAMP -positive samples were all confirmed to be BRV by sequencing the RT-LAMP- products restriction analysis of VP6 genes.

## Results

### The optimal protocol of RT-LAMP assay and inspection of products

**Figure 3:**
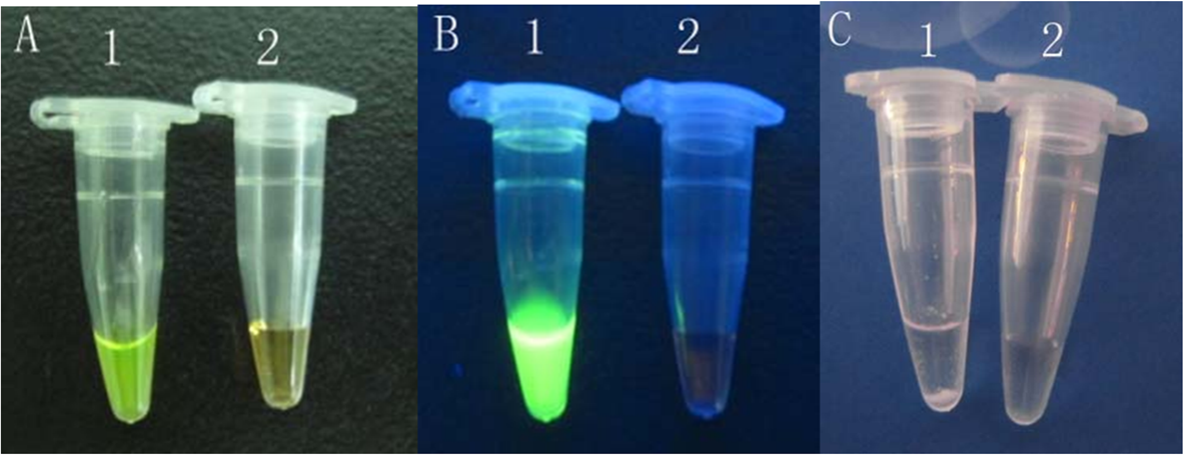
**Detection of BRV RT-LAMP product.A**: Fluorescent dye added seen without ultraviolet light, **B**: Fluorescent dye added seen with ultraviolet light, **C**: By turbidity with white sediment. 1: positive control sample; 2: negative control sample.

**Figure 4:**
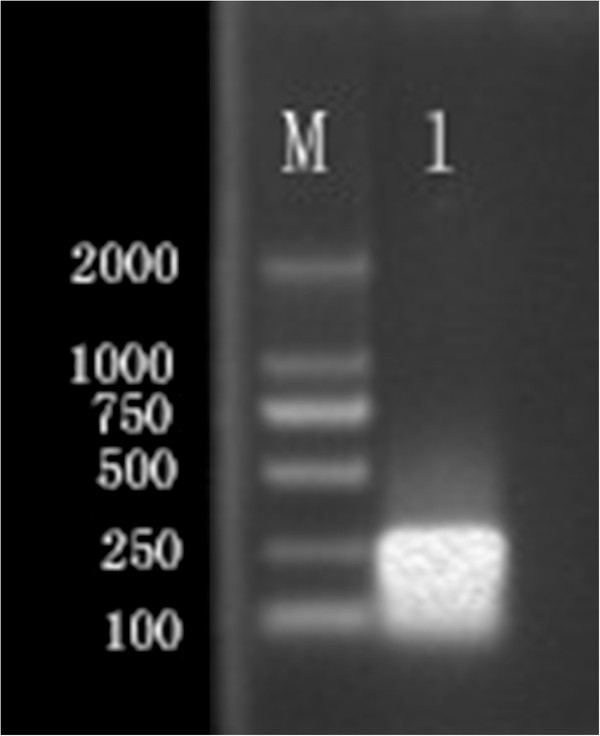
**Restriction analysis of RT-RT-LAMP product.** M. DNA marker; 1. RT-LAMP products digested with EcoRIand EcoRV.

Following standardization and optimization, the optimal ratio of primer (inner-outer-loop) concentrations for the RT-LAMP reaction was found to be 8:1:4 equivalents to 1.6, 0.2 and 0.8 mM. Gene amplification was detected by an increase in turbidity, as well as adding dye for color change indication (Figure 3). Restriction enzyme analysis performed with EcoRI and EcoRV on the RT-LAMP product validated no nonspecific reaction in RT-LAMP assay. The RT-LAMP assay amplified the 199-bp target sequence of the VP6 gene of BRV (Figure 4). For the reproducible sensitive and specific results of RT-LAMP assay, the optimal reaction time and incubation temperature was found to be 60 minutes at 63°C.

### Specificity and Sensitivity of RT-LAMP assay

**Figure 5:**
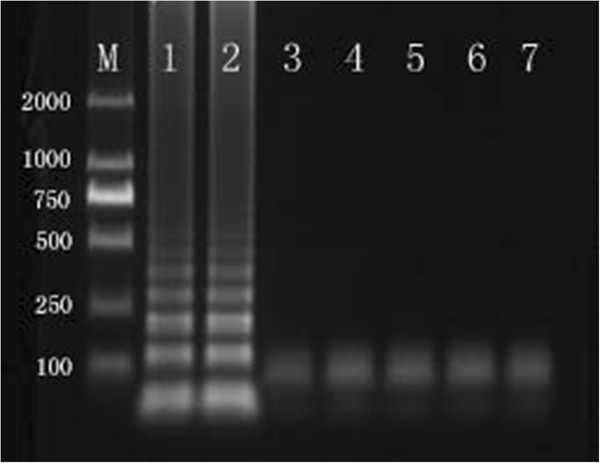
The detection of RT-LAMP product assessed by 1% agarose gel electrophoresis, M:DNA marker; 1: 014 strain, 2: NCDV strain, 3: BVDV, 4:IBRV, 5:CSFV, 6:MB, 7:BCV.

The bovine rotavirus-specific RT-LAMP assay specifically amplified strains NCDV-014, and 8 Guangxi field bovine rotavirus strains, which have been isolated from the Guangxi dairy farms, and exhibits no cross-reactivity with other pathogens (Table 1). This specificity was confirmed by agarose gel electrophoresis (Figure 5) and a color change assay (Figure 3). We also determined the assay sensitivity using a 10-fold dilution series. The detection limit of RT-LAMP was 3.32 copies (Figure 2-A). Similarly, the detection limit of real-time RT-PCR analysis was 3.32 copies (Figure 2-B). The results indicate that RT-LAMP is as sensitive as real-time RT-PCR, both of which can detect 3.32 copies of bovine rotavirus VP6 gene.

### Evaluation of RT-LAMP assay with clinical samples

In comparison with real-time RT-PCR, using the above described procedure with specific primers, 29 rectal swab samples (33.0%) were found positive by both real-time RT-PCR analysis and RT-LAMP, and 59 (70%) were found negative by both tests. As such, the coincidence of real-time RT-PCR and RT-LAMP was 100%. However, RT-LAMP is a quicker, easier, and more cost efficient method than real-time RT-PCR. Restriction enzyme revealed 199-bp target sequence of the VP6 gene of BRV, and sequencing analysis results showed that the clonal sequences of 29 samples were VP6 of BRV. The results indicated no nonspecific amplification in RT-LAMP reaction occurred (data not shown).

## Discussion

The RT-LAMP was shown here to be specific and sensitive to detect rotavirus in rectal swab samples of calves with acute diarrhea. The BRV-specific RT-LAMP primers and real time probe were designed using VP6 genes sequencing of 21 BRV isolates (Figure 1 a &b) from the Genbank data. VP6 a group-specific gene of BRV is the most immunogenic viral protein and considered to be very conserved among group A BRV and suitable to use as conserved gene specific for BRV [21-23]. The RT-LAMP was determined to be specific, as no cross-reactions were observed when other bovine pathogens were tested.

The RT-LAMP technique offers several advantages over PCR for the detection of bovine rotavirus RNA. Firstly, the RT-LAMP demonstrates high efficiency under isothermal conditions without a significant influence of non-target DNA and demonstrates sensitivity identical to that of real-time RT-PCR assays. Secondly, the RT- LAMP is easy to perform once the appropriate primers are selected and optimized. The reaction requires only four primers, a *Bst* DNA polymerase, and a temperature adjustable conventional laboratory water bath. Thirdly, the results of RT-LAMP are visible to the naked eye without the need for electrophoresis. This is attributable to recognition of the target sequence by six primers in the initial stage and by four primers during the later stages of the RT-LAMP reaction. The RT-LAMP system possesses specificity in addition to high sensitivity and may be used in large-scale molecular surveys of BRV infections in the field.

Nevertheless, RT-LAMP is not without caveats: First, the main challenge in development of molecular tests for BRV is the large genetic heterogeneity of this virus [3.4]. This emphasizes the need for constant updating of primers of molecular diagnostic tests for BRV, with FIP and BIP being the most important primers in the entire assay, and they need to be conserved across many strains. Second, due to its high amplification production, the issue of potential cross-contamination problems needs to be addressed. We have added dye into the reaction system before the actual amplification in order to eliminate contamination.

It should be pointed out that in utilizing theVP6 gene target for the optimization of specific primers, we would expect this BRV RT-LAMP assay to meet the practical needs of BRV detection in the field around the world. While the RT-LAMP developed here needs to be validated against rotaviruses of groups B and C and other groups, these bovine rotavirus groups have not yet been identified in cattle in southern China. To the authors’ knowledge, this is the first study that has explored the use of RT-LAMP technology in a diagnostic test for BRV. The test was simple, specific, and rapid; it was able to detect BRV from rectal swabs from BRV-infected calves.

## Conclusions

In this study, the established RT-LAMP assay with high sensitivity and specificity was performed in a water bath within only 60 minutes, and the amplification results were visualized by the naked eye after adding a fluorescent reagent. The newly developed assay can be used as one important tool for detecting BRV in calves under field conditions with no need for specialized equipment.

## Competing interests

The authors declare that they have no competing interests.

## Authors’ contributions

ZXX, QF and MIK designed the experiments. QF and LJX prepared the RNA samples. QF designed the primers and optimized conditions of the RT-LAMP assay. JBL, YSP, XWD and ZQX carried out the experiments shown in Figures 3, 4, 5 and 2 and in Table 1. 2, 3.4 QF and MIK performed the data analysis and wrote the manuscript. All authors read and approved the final manuscript.

## Supplementary Material

Additional file 1 Figure S1 and S2. Click here for file
