# Supplementary material for: Reverse transcription loop-mediated isothermal amplification assay for rapid detection of Bovine Rotavirus
Source: BMC Vet Res. 2012 Aug 15;8:133. doi: 10.1186/1746-6148-8-133 (PMC3599620; doi:10.1186/1746-6148-8-133)
Supplement: Additional file 1 — Figure S1 and S2. [file 1746-6148-8-133-S1.doc]

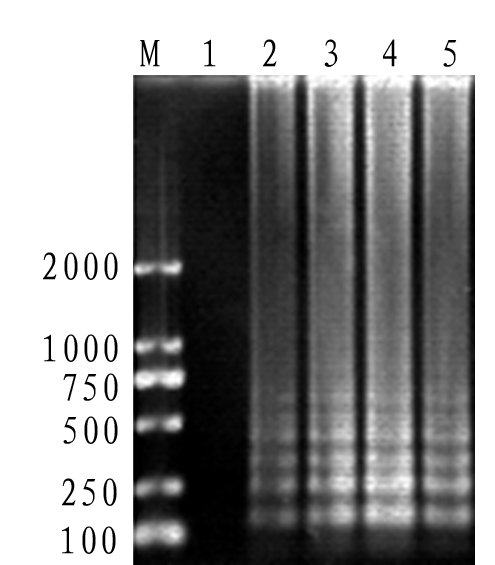


**Fig.1 Effect of temperature on LAMP reaction on gel electrophoresis**

M:DL2000 DNA Marker;1: Negative control 2:61℃;3:62℃;4:63℃;5: 64℃

Sample # 4 is brighter and the DNA bands are clearly visible so this could be optimized temperature


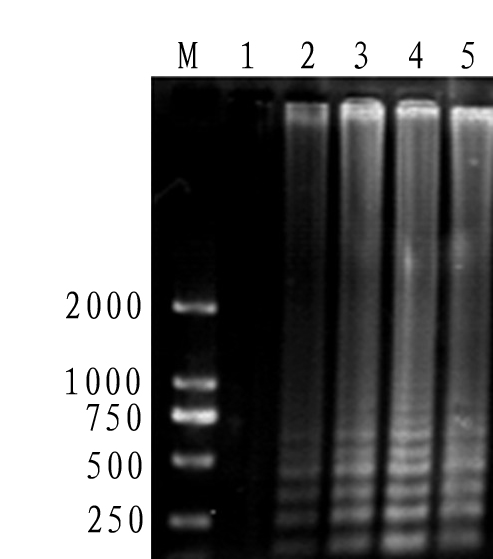


Fig 2. **Effect of time for LAMP reaction on gel electrophoresis**

M: DL2000 DNA Marker;1: Negative control 2:20 min;3:40 min;4:60 min;5: 80min

Sample # 4 the band are brighter and clearly visible could be the best for duration of incubation time.
